# Supplementary material for: Multiomics Data Collection, Visualization, and Utilization for Guiding Metabolic Engineering
Source: Front Bioeng Biotechnol. 2021 Feb 9;9:612893. doi: 10.3389/fbioe.2021.612893 (PMC7902046; doi:10.3389/fbioe.2021.612893)
Supplement: Supplementary file 11 [file Data_Sheet_2.ZIP › NotebookD.html]

D\_ART\_recommendations


# Notebook D: Generate recommendations for Cycle 2 using ART¶

This notebook uses the designs created by ART and the corresponding isoprenol production levels generated through OMG, to build a predictive model through ART. ART's model will be able to predict isoprenol production given the design as input. We will then use this model to recommend designs that optimize isoprenol production.

Tested using **ART\_v3.6** kernel on jprime.lbl.gov

## Inputs and Outputs¶

#### Required files to run this notebook:¶

- None, data will be automatically drawn from EDD

#### Files generated by running this notebook:¶

- `ART_training_EDDstyle.csv`, a file for ART front end import
- Standard ART output files (containing recommendations, predictive accuracy metrics, plots of predictions vs observations for the train data set, pickle file containing previously trained ART object) in the corresponding directory.
- `ARTrecommendations.csv`, a file containing the ART recommendations

## Setup¶

In [1]:

```
from edd_utils import login, export_study
```

## Downloading the data from EDD¶

First let's decide which study we want to get, from which EDD server, and using which login name:

In [2]:

```
study_slug = 'multiomics-be-strains-data-089b'
edd_server = 'public-edd.agilebiofoundry.org'
user       = 'tradivojevic'
```

Export the EDD study that contains isoprenol production data using `edd-utils` package (use your own username for EDD):

In [3]:

```
session = login(edd_server=edd_server, user=user)
```

In [4]:

```
df = export_study(session, study_slug, edd_server=edd_server)
df.head()
```

```

```

Out[4]:

|  | Study ID | Study Name | Line ID | Line Name | Line Description | Protocol | Assay ID | Assay Name | Formal Type | Measurement Type | Compartment | Units | Value | Hours |
| --- | --- | --- | --- | --- | --- | --- | --- | --- | --- | --- | --- | --- | --- | --- |
| 0 | 10114 | Multiomics BE strains data | 10115 | Strain 1 | ACCOAC\_1.0\_MDH\_1.0\_PTAr\_2.0\_CS\_0.0\_ACACT1r\_2.0... | Metabolomics | 10261 | Strain 1 | cid:12988 | 3-METHYL-3-BUTEN-1-OL | 0 | mM | 0.00000 | 9.0 |
| 1 | 10114 | Multiomics BE strains data | 10116 | Strain 2 | ACCOAC\_1.0\_MDH\_2.0\_PTAr\_2.0\_CS\_2.0\_ACACT1r\_2.0... | Metabolomics | 10247 | Strain 2 | cid:12988 | 3-METHYL-3-BUTEN-1-OL | 0 | mM | 0.55210 | 9.0 |
| 2 | 10114 | Multiomics BE strains data | 10117 | Strain 3 | ACCOAC\_1.0\_MDH\_0.0\_PTAr\_0.0\_CS\_2.0\_ACACT1r\_1.0... | Metabolomics | 10277 | Strain 3 | cid:12988 | 3-METHYL-3-BUTEN-1-OL | 0 | mM | 0.34920 | 9.0 |
| 3 | 10114 | Multiomics BE strains data | 10118 | Strain 4 | ACCOAC\_1.0\_MDH\_1.0\_PTAr\_1.0\_CS\_1.0\_ACACT1r\_2.0... | Metabolomics | 10281 | Strain 4 | cid:12988 | 3-METHYL-3-BUTEN-1-OL | 0 | mM | 0.55185 | 9.0 |
| 4 | 10114 | Multiomics BE strains data | 10119 | Strain 5 | ACCOAC\_2.0\_MDH\_0.0\_PTAr\_2.0\_CS\_1.0\_ACACT1r\_1.0... | Metabolomics | 10292 | Strain 5 | cid:12988 | 3-METHYL-3-BUTEN-1-OL | 0 | mM | 0.08012 | 9.0 |

Keep only the necesarry columns:

In [5]:

```
df = df[['Line Name','Line Description','Measurement Type', 'Value']]
df.head()
```

Out[5]:

|  | Line Name | Line Description | Measurement Type | Value |
| --- | --- | --- | --- | --- |
| 0 | Strain 1 | ACCOAC\_1.0\_MDH\_1.0\_PTAr\_2.0\_CS\_0.0\_ACACT1r\_2.0... | 3-METHYL-3-BUTEN-1-OL | 0.00000 |
| 1 | Strain 2 | ACCOAC\_1.0\_MDH\_2.0\_PTAr\_2.0\_CS\_2.0\_ACACT1r\_2.0... | 3-METHYL-3-BUTEN-1-OL | 0.55210 |
| 2 | Strain 3 | ACCOAC\_1.0\_MDH\_0.0\_PTAr\_0.0\_CS\_2.0\_ACACT1r\_1.0... | 3-METHYL-3-BUTEN-1-OL | 0.34920 |
| 3 | Strain 4 | ACCOAC\_1.0\_MDH\_1.0\_PTAr\_1.0\_CS\_1.0\_ACACT1r\_2.0... | 3-METHYL-3-BUTEN-1-OL | 0.55185 |
| 4 | Strain 5 | ACCOAC\_2.0\_MDH\_0.0\_PTAr\_2.0\_CS\_1.0\_ACACT1r\_1.0... | 3-METHYL-3-BUTEN-1-OL | 0.08012 |

### Adding design information to the data frame¶

Add columns for each reaction:

In [6]:

```
reactions = df['Line Description'][0].split('_')[::2]
for rxn in reactions:
    df[rxn] = None

df.tail()
```

Out[6]:

|  | Line Name | Line Description | Measurement Type | Value | ACCOAC | MDH | PTAr | CS | ACACT1r | PPC | PPCK | PFL |
| --- | --- | --- | --- | --- | --- | --- | --- | --- | --- | --- | --- | --- |
| 91 | Strain 92 | ACCOAC\_0.0\_MDH\_0.0\_PTAr\_2.0\_CS\_2.0\_ACACT1r\_0.0... | 3-METHYL-3-BUTEN-1-OL | 0.00000 | None | None | None | None | None | None | None | None |
| 92 | Strain 93 | ACCOAC\_1.0\_MDH\_2.0\_PTAr\_0.0\_CS\_0.0\_ACACT1r\_0.0... | 3-METHYL-3-BUTEN-1-OL | 0.00000 | None | None | None | None | None | None | None | None |
| 93 | Strain 94 | ACCOAC\_0.0\_MDH\_2.0\_PTAr\_0.0\_CS\_1.0\_ACACT1r\_0.0... | 3-METHYL-3-BUTEN-1-OL | 0.00000 | None | None | None | None | None | None | None | None |
| 94 | Strain 95 | ACCOAC\_0.0\_MDH\_1.0\_PTAr\_2.0\_CS\_0.0\_ACACT1r\_1.0... | 3-METHYL-3-BUTEN-1-OL | 0.00000 | None | None | None | None | None | None | None | None |
| 95 | WT | Wild type E. coli | 3-METHYL-3-BUTEN-1-OL | 0.46188 | None | None | None | None | None | None | None | None |

And assign values for each reaction and line:

In [7]:

```
for i in range(len(df)):
    if df['Line Name'][i]=='WT':
        for r in range(len(reactions)):
            df.iloc[i, (4+r)] = float(1)
    else:
        values = df.loc[i]['Line Description'].split('_')[1::2]
        for r,value in zip(range(len(reactions)),values):
            df.iloc[i, (4+r)] = float(value)

df = df.drop(columns='Line Description')
```

Each design (line) involves the modification of up to 8 fluxes (1 -> keep the same; 2-> double flux, 0-> knock reaction out):

In [8]:

```
df.tail()
```

Out[8]:

|  | Line Name | Measurement Type | Value | ACCOAC | MDH | PTAr | CS | ACACT1r | PPC | PPCK | PFL |
| --- | --- | --- | --- | --- | --- | --- | --- | --- | --- | --- | --- |
| 91 | Strain 92 | 3-METHYL-3-BUTEN-1-OL | 0.00000 | 0 | 0 | 2 | 2 | 0 | 2 | 0 | 2 |
| 92 | Strain 93 | 3-METHYL-3-BUTEN-1-OL | 0.00000 | 1 | 2 | 0 | 0 | 0 | 2 | 2 | 0 |
| 93 | Strain 94 | 3-METHYL-3-BUTEN-1-OL | 0.00000 | 0 | 2 | 0 | 1 | 0 | 2 | 1 | 1 |
| 94 | Strain 95 | 3-METHYL-3-BUTEN-1-OL | 0.00000 | 0 | 1 | 2 | 0 | 1 | 1 | 0 | 2 |
| 95 | WT | 3-METHYL-3-BUTEN-1-OL | 0.46188 | 1 | 1 | 1 | 1 | 1 | 1 | 1 | 1 |

How many designs improve production over the wild type?

In [9]:

```
num_improved_production = len(df[df['Value'] > df.loc[95]['Value']])
print(f'{num_improved_production} designs out of {len(df)} improve production of isoprenol ({num_improved_production/len(df)*100:.2f}%).')
```

```
11 designs out of 96 improve production of isoprenol (11.46%).
```

Rename `Value` column to the formal metabolite name:

In [10]:

```
production_name = df['Measurement Type'][0]
df = df.rename(columns={'Value': production_name})
df = df.drop(columns='Measurement Type')
df.tail()
```

Out[10]:

|  | Line Name | 3-METHYL-3-BUTEN-1-OL | ACCOAC | MDH | PTAr | CS | ACACT1r | PPC | PPCK | PFL |
| --- | --- | --- | --- | --- | --- | --- | --- | --- | --- | --- |
| 91 | Strain 92 | 0.00000 | 0 | 0 | 2 | 2 | 0 | 2 | 0 | 2 |
| 92 | Strain 93 | 0.00000 | 1 | 2 | 0 | 0 | 0 | 2 | 2 | 0 |
| 93 | Strain 94 | 0.00000 | 0 | 2 | 0 | 1 | 0 | 2 | 1 | 1 |
| 94 | Strain 95 | 0.00000 | 0 | 1 | 2 | 0 | 1 | 1 | 0 | 2 |
| 95 | WT | 0.46188 | 1 | 1 | 1 | 1 | 1 | 1 | 1 | 1 |

In [11]:

```
df[df[production_name] > df.loc[95][production_name]]
```

Out[11]:

|  | Line Name | 3-METHYL-3-BUTEN-1-OL | ACCOAC | MDH | PTAr | CS | ACACT1r | PPC | PPCK | PFL |
| --- | --- | --- | --- | --- | --- | --- | --- | --- | --- | --- |
| 1 | Strain 2 | 0.55210 | 1 | 2 | 2 | 2 | 2 | 1 | 1 | 0 |
| 3 | Strain 4 | 0.55185 | 1 | 1 | 1 | 1 | 2 | 2 | 1 | 0 |
| 6 | Strain 7 | 0.57265 | 2 | 1 | 2 | 2 | 2 | 2 | 0 | 2 |
| 16 | Strain 17 | 0.49510 | 2 | 2 | 0 | 2 | 2 | 2 | 0 | 2 |
| 40 | Strain 41 | 0.57265 | 1 | 2 | 1 | 2 | 1 | 1 | 1 | 2 |
| 45 | Strain 46 | 0.57265 | 2 | 2 | 2 | 2 | 2 | 1 | 1 | 1 |
| 47 | Strain 48 | 0.57265 | 2 | 1 | 1 | 2 | 1 | 2 | 1 | 1 |
| 48 | Strain 49 | 0.57265 | 1 | 2 | 1 | 2 | 2 | 1 | 0 | 1 |
| 61 | Strain 62 | 0.55273 | 1 | 2 | 1 | 2 | 1 | 2 | 0 | 0 |
| 66 | Strain 67 | 0.57265 | 2 | 2 | 1 | 1 | 1 | 1 | 0 | 1 |
| 79 | Strain 80 | 0.49642 | 1 | 1 | 0 | 2 | 1 | 1 | 1 | 0 |

### Saving the data in EDD format, for use with the front end.¶

Pivot the dataframe back to EDD format, now including all the reaction names and modifications:

In [12]:

```
df = df.set_index('Line Name').stack().reset_index()
df.columns = ['Line Name', 'Measurement Type', 'Value']
df.head()
```

Out[12]:

|  | Line Name | Measurement Type | Value |
| --- | --- | --- | --- |
| 0 | Strain 1 | 3-METHYL-3-BUTEN-1-OL | 0.0 |
| 1 | Strain 1 | ACCOAC | 1.0 |
| 2 | Strain 1 | MDH | 1.0 |
| 3 | Strain 1 | PTAr | 2.0 |
| 4 | Strain 1 | CS | 0.0 |

Save this dataframe to a file for ART front end:

In [13]:

```
data_file = '../data/ART_training_EDDstyle.csv'
df.to_csv(data_file, header=True, index=False)
```

Store the names of all variables:

In [14]:

```
variables = df['Measurement Type'][df['Line Name']=='Strain 1'].tolist()
```

# Running ART to create a predictive model of production and generate recommendations¶

The first step is to make sure the ART library is available in your kernel (ART\_v3.6 has all the necessary dependencies). Clone the corresponding git repository:

`git clone https://github.com/JBEI/AutomatedRecommendationTool.git`

(Information about licensing ART is available at https://github.com/JBEI/ART.)

We can then add library to the path and do the necessary imports:

In [15]:

```
import sys
sys.path.append('../../AutomatedRecommendationTool')
    
from art.core import *
import pickle
```

And then define some ART input parameters:

In [16]:

```
user_params = {}
user_params['num_recommendations'] = 10                  # Number of final recommendations
user_params['output_directory'] = '../data/art_output/'  # Directory to store output files
```

## Run ART¶

The first step is to create a dictionary that contains the settings for ART:

In [17]:

```
art_params = {
    'response_var': [variables[0]],
    'input_var': variables[1:],
    'input_var_type': 'Categorical',
    'seed': 10,
    'num_recommendations': user_params['num_recommendations'],   
    'cross_val': True,
    'output_directory': user_params['output_directory']
}
```

With this setting, you can now run ART. However, this takes around 25min, so you can set run\_art to False and load the previously run model, which will be much faster:

In [18]:

```
run_art = True
```

The folliwing cell will generate plots of the (cross-validated) predictions vs observation, to gauge the quality of the predictions; a plot of the predicted distribution for all recommendations; a plot of success probability (for improving the current best production) vs the number of recommended strains engineered.

In [19]:

```
%%time
if run_art:
    art = RecommendationEngine(df, **art_params)
else:
    with open(os.path.join(art_params['output_directory'], 'art.pkl'), 'rb') as output:
        art = pickle.load(output)
```

```
Warning: {warn}
Warning: xgboost.XGBRegressor is not available and will not be used by TPOT.
```

```
Multiprocess sampling (2 chains in 2 jobs)
NUTS: [omegas, sigma]
There were 2 divergences after tuning. Increase `target_accept` or reparameterize.
```

```
Warning: xgboost.XGBRegressor is not available and will not be used by TPOT.
```

```
Multiprocess sampling (2 chains in 2 jobs)
NUTS: [omegas, sigma]
There were 2 divergences after tuning. Increase `target_accept` or reparameterize.
The acceptance probability does not match the target. It is 0.6595131800190912, but should be close to 0.8. Try to increase the number of tuning steps.
There were 12 divergences after tuning. Increase `target_accept` or reparameterize.
```

```
Warning: xgboost.XGBRegressor is not available and will not be used by TPOT.
```

```
Multiprocess sampling (2 chains in 2 jobs)
NUTS: [omegas, sigma]
There was 1 divergence after tuning. Increase `target_accept` or reparameterize.
There were 12 divergences after tuning. Increase `target_accept` or reparameterize.
The acceptance probability does not match the target. It is 0.6462472422719335, but should be close to 0.8. Try to increase the number of tuning steps.
```

```
Warning: xgboost.XGBRegressor is not available and will not be used by TPOT.
```

```
Multiprocess sampling (2 chains in 2 jobs)
NUTS: [omegas, sigma]
There were 11 divergences after tuning. Increase `target_accept` or reparameterize.
There were 2 divergences after tuning. Increase `target_accept` or reparameterize.
```

```
Warning: xgboost.XGBRegressor is not available and will not be used by TPOT.
```

```
Multiprocess sampling (2 chains in 2 jobs)
NUTS: [omegas, sigma]
There were 2 divergences after tuning. Increase `target_accept` or reparameterize.
There were 9 divergences after tuning. Increase `target_accept` or reparameterize.
```

```
Warning: xgboost.XGBRegressor is not available and will not be used by TPOT.
```

```
Multiprocess sampling (2 chains in 2 jobs)
NUTS: [omegas, sigma]
There were 11 divergences after tuning. Increase `target_accept` or reparameterize.
There were 2 divergences after tuning. Increase `target_accept` or reparameterize.
```

```
Warning: xgboost.XGBRegressor is not available and will not be used by TPOT.
```

```
Multiprocess sampling (2 chains in 2 jobs)
NUTS: [omegas, sigma]
There was 1 divergence after tuning. Increase `target_accept` or reparameterize.
The acceptance probability does not match the target. It is 0.9298139747992613, but should be close to 0.8. Try to increase the number of tuning steps.
There were 4 divergences after tuning. Increase `target_accept` or reparameterize.
```

```
Warning: xgboost.XGBRegressor is not available and will not be used by TPOT.
```

```
Multiprocess sampling (2 chains in 2 jobs)
NUTS: [omegas, sigma]
```

```
Warning: xgboost.XGBRegressor is not available and will not be used by TPOT.
```

```
Multiprocess sampling (2 chains in 2 jobs)
NUTS: [omegas, sigma]
There was 1 divergence after tuning. Increase `target_accept` or reparameterize.
```

```
Warning: xgboost.XGBRegressor is not available and will not be used by TPOT.
```

```
Multiprocess sampling (2 chains in 2 jobs)
NUTS: [omegas, sigma]
There were 4 divergences after tuning. Increase `target_accept` or reparameterize.
There were 3 divergences after tuning. Increase `target_accept` or reparameterize.
```

```
Warning: xgboost.XGBRegressor is not available and will not be used by TPOT.
```

```
Multiprocess sampling (2 chains in 2 jobs)
NUTS: [omegas, sigma]
There was 1 divergence after tuning. Increase `target_accept` or reparameterize.
There was 1 divergence after tuning. Increase `target_accept` or reparameterize.
```

```
CPU times: user 26min 6s, sys: 13min 51s, total: 39min 57s
Wall time: 22min 34s
```

In [20]:

```
utils.save_pkl_object(art)
```

### List the set of design recommendations generated by ART¶

In [21]:

```
art.recommendations
```

Out[21]:

|  | ACCOAC | MDH | PTAr | CS | ACACT1r | PPC | PPCK | PFL | 3-METHYL-3-BUTEN-1-OL |
| --- | --- | --- | --- | --- | --- | --- | --- | --- | --- |
| 0 | 2.0 | 2.0 | 1.0 | 2.0 | 2.0 | 2.0 | 0.0 | 1.0 | 0.573584 |
| 1 | 2.0 | 2.0 | 2.0 | 2.0 | 2.0 | 2.0 | 0.0 | 1.0 | 0.572193 |
| 2 | 1.0 | 2.0 | 1.0 | 2.0 | 2.0 | 2.0 | 0.0 | 1.0 | 0.572167 |
| 3 | 2.0 | 1.0 | 1.0 | 2.0 | 2.0 | 2.0 | 0.0 | 1.0 | 0.572039 |
| 4 | 2.0 | 2.0 | 1.0 | 2.0 | 2.0 | 1.0 | 0.0 | 1.0 | 0.571967 |
| 5 | 2.0 | 2.0 | 2.0 | 2.0 | 2.0 | 1.0 | 0.0 | 1.0 | 0.571282 |
| 6 | 2.0 | 2.0 | 1.0 | 2.0 | 2.0 | 2.0 | 0.0 | 2.0 | 0.570968 |
| 7 | 2.0 | 2.0 | 2.0 | 2.0 | 2.0 | 2.0 | 0.0 | 2.0 | 0.570915 |
| 8 | 1.0 | 2.0 | 2.0 | 2.0 | 2.0 | 2.0 | 0.0 | 1.0 | 0.570771 |
| 9 | 2.0 | 1.0 | 2.0 | 2.0 | 2.0 | 2.0 | 0.0 | 1.0 | 0.570769 |

It turns out that all recommendations indicate that the CS and ACACT1r reaction fluxes should double and PPCK should be knocked out.

### Save recommendations into a file¶

Finally we save the recommendations, along with the predicted production levels. We will compare them with the ground truth provided by OMG in the next notebook.

First, we change the last column name to indicate it is predicted:

In [23]:

```
pred_col_name = 'Mean predicted Isoprenol [mM]'
art.recommendations = art.recommendations.rename(columns={art_params['response_var'][0]: pred_col_name})
```

Then, we add standard deviation predictions for the recommendations:

In [24]:

```
pp_rec_mean, pp_rec_std = art.post_pred_stats(art.recommendations.values[:,:-1])
art.recommendations['SD Isoprenol [mM]'] = pp_rec_std.copy()
```

We assign Line Name to each of the recommendations:

In [25]:

```
n_instances = len(set(df['Line Name']))
art.recommendations.insert(loc=0, column='Line Name', value=['Strain ' + str(n_instances+i) for i in range(1,art_params['num_recommendations']+1)])
art.recommendations.head()
```

Out[25]:

|  | Line Name | ACCOAC | MDH | PTAr | CS | ACACT1r | PPC | PPCK | PFL | Mean predicted Isoprenol [mM] | SD Isoprenol [mM] |
| --- | --- | --- | --- | --- | --- | --- | --- | --- | --- | --- | --- |
| 0 | Strain 97 | 2.0 | 2.0 | 1.0 | 2.0 | 2.0 | 2.0 | 0.0 | 1.0 | 0.573584 | 0.019785 |
| 1 | Strain 98 | 2.0 | 2.0 | 2.0 | 2.0 | 2.0 | 2.0 | 0.0 | 1.0 | 0.572193 | 0.019752 |
| 2 | Strain 99 | 1.0 | 2.0 | 1.0 | 2.0 | 2.0 | 2.0 | 0.0 | 1.0 | 0.572167 | 0.019752 |
| 3 | Strain 100 | 2.0 | 1.0 | 1.0 | 2.0 | 2.0 | 2.0 | 0.0 | 1.0 | 0.572039 | 0.019787 |
| 4 | Strain 101 | 2.0 | 2.0 | 1.0 | 2.0 | 2.0 | 1.0 | 0.0 | 1.0 | 0.571967 | 0.019780 |

And finally save it in a file:

In [26]:

```
rec_filename = f'{art.outDir}/ARTrecommendations.csv'
art.recommendations.to_csv(rec_filename, header=True, index=False)
```

In [ ]:

```

```
