# Supplementary material for: Multiomics Data Collection, Visualization, and Utilization for Guiding Metabolic Engineering
Source: Front Bioeng Biotechnol. 2021 Feb 9;9:612893. doi: 10.3389/fbioe.2021.612893 (PMC7902046; doi:10.3389/fbioe.2021.612893)
Supplement: Supplementary file 11 [file Data_Sheet_2.ZIP › NotebookA.html]

A\_Generate\_omics\_data\_WT


# Notebook A: Generation of omics data for the wild type (WT) strain¶

This notebook uses the OMG library to create times series of synthetic "experimental" data (transcriptomics, proteomics, metabolomics, fluxomics, cell density, external metabolites), that will be used to demonstrate the use of ICE and EDD. These data will also be the base for creating similar data for bioengineereed strains.

Tested using **biodesign\_3.7** kernel on jprime.lbl.gov (see github repository for kernel details)

## Inputs and outputs¶

#### Required file to run this notebook:¶

- A modified E. coli model with the isoprenol pathway added to it (`iJO1366_MVA.json` file in the `../data/models` directory)

#### Files generated by running this notebook for import into EDD:¶

- `EDD_experiment_description_file_WT.csv`
- `EDD_OD_WT.csv`
- `EDD_external_metabolites_WT.csv`
- `EDD_transcriptomics_WT.csv`
- `EDD_proteomics_WTSM.csv`
- `EDD_metabolomics_WTSM.csv`
- `EDD_fluxomics_WT.csv`

The files are stored in the user defined directory.

## Setup¶

Clone the git repository with the `OMG` library:

`git clone https://github.com/JBEI/OMG.git`

or pull the latest version.

Importing needed libraries:

In [1]:

```
import sys

sys.path.insert(1, '../../OMG')
sys.path.append('../')

import omg 
from plot_multiomics import *
import cobra
```

## User parameters¶

In [2]:

```
user_params = {
    'host': 'ecoli',                                        # ecoli or ropacus supported
    'modelfile': '../data/models/iJO1366_MVA.json',         # GSM host model file location
    'cerevisiae_modelfile': '../data/models/iMM904.json',   # GSM pathway donor model file location
    'timestart': 0.0,                                       # Start and end for time in time series
    'timestop': 8.0,
    'numtimepoints': 9,                                     # Number of time points
    'mapping_file': '../mapping/inchikey_to_cid.txt',       # Maps of metabolite inchikey to pubchem compound id (cid) 
    'output_file_path': '../data/omg_output/',              # Folder for output files
    'edd_omics_file_path': '../data/omg_output/edd/',       # Folder for EDD output files
    'numreactions': 8,                                      # Number of total reactions to be bioengineered
    'ext_metabolites': {                                    # Initial concentrations (in mMol) of external metabolites
        'glc__D_e': 22.203,
        'nh4_e': 18.695,
        'pi_e': 69.454,
        'so4_e': 2.0,
        'mg2_e': 2.0,
        'k_e': 21.883,
        'na1_e': 103.7,
        'cl_e': 27.25,
        'isoprenol_e': 0.0,
        'ac_e': 0.0,
        'for_e': 0.0,
        'lac__D_e': 0.0,
        'etoh_e': 0.0
    },
    'initial_OD': 0.01,                                      
    'BIOMASS_REACTION_ID': 'BIOMASS_Ec_iJO1366_core_53p95M' # Biomass reaction in host GSM
}
```

## Using the OMG library to create synthetic multiomics data¶

### 1) Getting and preparing the metabolic model¶

First we obtain the metabolic model:

In [3]:

```
file_name = user_params['modelfile']
model = cobra.io.load_json_model(file_name)
```

We now add minimum flux constraints for production of isoprenol and formate, and we limit oxygen intake:

In [4]:

```
iso = 'EX_isoprenol_e'
iso_cons = model.problem.Constraint(model.reactions.EX_isoprenol_e.flux_expression,lb = 0.20)
model.add_cons_vars(iso_cons)
for_cons = model.problem.Constraint(model.reactions.EX_for_e.flux_expression,lb = 0.10)
model.add_cons_vars(for_cons)
o2_cons = model.problem.Constraint(model.reactions.EX_o2_e.flux_expression,lb = -8.0)
model.add_cons_vars(o2_cons)
```

And then we constrain several central carbon metabolism fluxes to more realistic upper and lower bounds:

In [5]:

```
CC_rxn_names = ['ACCOAC','MDH','PTAr','CS','ACACT1r','PPC','PPCK','PFL']
for reaction in CC_rxn_names:
    reaction_constraint = model.problem.Constraint(model.reactions.get_by_id(reaction).flux_expression,lb = -1.0,ub =  1.0)
    model.add_cons_vars(reaction_constraint)
```

### 2) Obtaining fluxomics times series¶

First create time grid for simulation:

In [6]:

```
t0 = user_params['timestart']
tf = user_params['timestop']
points = user_params['numtimepoints']
tspan, delt = np.linspace(t0, tf, points, dtype='float64', retstep=True)

grid = (tspan, delt)
```

We then use this model to obtain the times series for fluxes, OD and external metabolites, by solving the model for each time point:

In [7]:

```
solution_TS, model_TS, cell, Emets, Erxn2Emet = \
    omg.get_flux_time_series(model, user_params['ext_metabolites'], grid, user_params)
```

```
0.0 optimal 0.5363612610171437
1.0 optimal 0.5363612610171437
2.0 optimal 0.5363612610171437
3.0 optimal 0.5363612610171437
4.0 optimal 0.5363612610171437
5.0 optimal 0.5363612610171437
6.0 optimal 0.5363612610171437
7.0 optimal 0.5363612610171437
8.0 optimal 0.5363612610171437
```

These are the external metabolites concentrations as a function of time:

In [8]:

```
Emets
```

Out[8]:

|  | glc\_\_D\_e | nh4\_e | pi\_e | so4\_e | mg2\_e | k\_e | na1\_e | cl\_e | isoprenol\_e | ac\_e | for\_e | lac\_\_D\_e | etoh\_e |
| --- | --- | --- | --- | --- | --- | --- | --- | --- | --- | --- | --- | --- | --- |
| 0.0 | 22.0707 | 18.6183 | 69.4472 | 1.99821 | 1.99994 | 21.8816 | 103.7 | 27.25 | 0.00264663 | 0.027405 | 0.00132331 | 0.102538 | 0 |
| 1.0 | 21.8444 | 18.4873 | 69.4354 | 1.99515 | 1.99983 | 21.8792 | 103.7 | 27.2499 | 0.00717176 | 0.0742613 | 0.00358588 | 0.277855 | 0 |
| 2.0 | 21.4576 | 18.2632 | 69.4154 | 1.98992 | 1.99965 | 21.8752 | 103.7 | 27.2498 | 0.0149087 | 0.154375 | 0.00745436 | 0.577608 | 0 |
| 3.0 | 20.7961 | 17.88 | 69.3812 | 1.98097 | 1.99935 | 21.8683 | 103.7 | 27.2496 | 0.0281372 | 0.291351 | 0.0140686 | 1.09012 | 0 |
| 4.0 | 19.6653 | 17.2248 | 69.3227 | 1.96567 | 1.99882 | 21.8564 | 103.7 | 27.2493 | 0.0507548 | 0.52555 | 0.0253774 | 1.96639 | 0 |
| 5.0 | 17.7317 | 16.1047 | 69.2227 | 1.93951 | 1.99792 | 21.8362 | 103.7 | 27.2488 | 0.0894259 | 0.925977 | 0.044713 | 3.46462 | 0 |
| 6.0 | 14.4258 | 14.1895 | 69.0516 | 1.89479 | 1.99638 | 21.8016 | 103.7 | 27.2478 | 0.155545 | 1.61062 | 0.0777724 | 6.02626 | 0 |
| 7.0 | 8.77335 | 10.915 | 68.7592 | 1.81833 | 1.99375 | 21.7424 | 103.7 | 27.2463 | 0.268593 | 2.7812 | 0.134297 | 10.4061 | 0 |
| 8.0 | 0 | 5.31631 | 68.2591 | 1.68759 | 1.98925 | 21.6412 | 103.7 | 27.2436 | 0.46188 | 4.78262 | 0.23094 | 17.8946 | 0 |
| 9.0 | 0 | 5.31631 | 68.2591 | 1.68759 | 1.98925 | 21.6412 | 103.7 | 27.2436 | 0.46188 | 4.78262 | 0.23094 | 17.8946 | 0 |

In [9]:

```
plot_DO_extmets(cell, Emets[['glc__D_e','isoprenol_e','ac_e','for_e','lac__D_e','etoh_e']])
```

### 3) Use fluxomics data to obtain the rest of multiomics data¶

We now obtain the multiomics data for each time point:

In [10]:

```
proteomics_timeseries = {}
transcriptomics_timeseries = {}
metabolomics_timeseries = {}
metabolomics_oldids_timeseries = {}
fluxomics_timeseries = {}
        
# By setting the old_ids flag to True, we get two time series for metabolomics data: one with Pubchem CIDs and one with BIGG ids.
# Setting the old_ids flag to False and returns only three dictionaries:proteomics, transcriptomics, metabolomics

for t in tspan:
    fluxomics_timeseries[t] = solution_TS[t].fluxes.to_dict()
    (proteomics_timeseries[t], transcriptomics_timeseries[t], 
     metabolomics_timeseries[t], metabolomics_oldids_timeseries[t]) = omg.get_multiomics(model, 
                                                                                         solution_TS[t], 
                                                                                         user_params['mapping_file'], 
                                                                                         old_ids=True)
```

### 4) Write the multiomics, cell concentration and external metabolites data into output files¶

#### EDD data output¶

First write the experiment description files needed for input (label indicates a label at the end of the file name):

In [11]:

```
omg.write_experiment_description_file(user_params['edd_omics_file_path'], line_name='WT', label='_WT')
```

Write OD data:

In [12]:

```
omg.write_OD_data(cell, user_params['edd_omics_file_path'], line_name='WT', label='_WT')
```

Write external metabolites:

In [13]:

```
omg.write_external_metabolite(Emets, user_params['edd_omics_file_path'], line_name='WT', label='_WT')
```

Write multiomics data:

In [14]:

```
omg.write_omics_files(fluxomics_timeseries, 'fluxomics', user_params, line_name='WT', label='_WT')
omg.write_omics_files(proteomics_timeseries, 'proteomics', user_params, line_name='WT', label='_WT')
omg.write_omics_files(transcriptomics_timeseries, 'transcriptomics', user_params, line_name='WT', label='_WT')
omg.write_omics_files(metabolomics_timeseries, 'metabolomics', user_params, line_name='WT', label='_WT')
```

We will also write a small version of the multiomics data with a subset of proteins, transcripts and metabolites:

In [15]:

```
genesSM       = ['b0180','b2708','b3197','b1094','b2224','b3256','b2316','b3255','b0185','b1101'] 
proteinsSM    = ['P17115','P76461','P0ABD5','P00893','P15639','P0AC44','P0A6I6','P0A9M8']
metabolitesSM = ['CID:1549101','CID:175','CID:164533','CID:15938965','CID:21604863','CID:15939608','CID:27284','CID:1038','CID:16741146','CID:1778309']
```

In [16]:

```
transcriptomics_timeseriesSM ={}
proteomics_timeseriesSM      ={}
metabolomics_timeseriesSM    ={}
for t in tspan:
    transcriptomics_timeseriesSM[t] = {gene:    transcriptomics_timeseries[t][gene] for gene    in genesSM}
    proteomics_timeseriesSM[t]      = {protein: proteomics_timeseries[t][protein]   for protein in proteinsSM}
    metabolomics_timeseriesSM[t]    = {metab:   metabolomics_timeseries[t][metab]   for metab   in metabolitesSM}
```

In [17]:

```
omg.write_omics_files(proteomics_timeseriesSM,     'proteomics'     , user_params, line_name='WT', label='_WTSM')
omg.write_omics_files(transcriptomics_timeseriesSM,'transcriptomics', user_params, line_name='WT', label='_WTSM')
omg.write_omics_files(metabolomics_timeseriesSM,   'metabolomics'   , user_params, line_name='WT', label='_WTSM')
```

In [ ]:

```

```
