# Supplementary material for: Multiomics Data Collection, Visualization, and Utilization for Guiding Metabolic Engineering
Source: Front Bioeng Biotechnol. 2021 Feb 9;9:612893. doi: 10.3389/fbioe.2021.612893 (PMC7902046; doi:10.3389/fbioe.2021.612893)
Supplement: Supplementary file 11 [file Data_Sheet_2.ZIP › NotebookB.html]

B\_Generate\_initial\_designs


# Notebook B: Generate initial designs for training data for ART¶

This notebook designs the experiment needed to feed ART's predictive capabilities. We use ART to provide suggested designs (flux modifications) for which to get phenotypic data. These designs and phenotypic data will be used later to predict new designs.

Tested using **ART\_v3.6** kernel on jprime.lbl.gov

## Inputs and output¶

#### No required files to run this notebook¶

#### File generated by running this notebook¶

- `ICE_MOstrains.csv` (to be used for ICE import)

## Setup¶

Clone the git repository with the `ART` library

`git clone https://github.com/JBEI/AutomatedRecommendationTool.git`

or pull the latest version.

Information about licensing ART is available at https://github.com/JBEI/ART.

Importing needed libraries:

In [1]:

```
import sys
sys.path.append('../../AutomatedRecommendationTool')        # Make sure this is the location for the ART library 
sys.path.append('../')
    
from art.core import *                                  
from plot_multiomics import plot_distribution_of_designs
```

## User parameters¶

### Defining fluxes to be modified and the number of instances (designs) to be created¶

These are the reactions we consider for genetic engineering in terms of isoprenol production:

In [2]:

```
user_params = {
    'reactions': ['ACCOAC', 
                  'MDH',
                  'PTAr', 
                  'CS', 
                  'ACACT1r', 
                  'PPC', 
                  'PPCK',
                  'PFL']
}
```

In [3]:

```
n_reactions = len(user_params['reactions'])
```

We consider the following possible genetic modifications:

- knockout (KO)
- no modification (NoMod)
- upregulation 2x (UP)

and we describe them through numerical categories:

In [4]:

```
user_params['modif_dict'] = {
    'KO': int(0), 
    'NoMod': int(1),
    'UP': int(2)
}
```

Here we specify how many instances (designs) we want to create (change as desired):

In [5]:

```
user_params['n_instances'] = 96
```

And we define the path and name for the output file:

In [6]:

```
user_params['designs_ice_file'] = '../data/art_output/ICE_MOstrains.csv'
```

### Calculating the size of the full design space and relative coverage¶

Here we calculate which percentage of possible designs we have covered with this initial set:

In [7]:

```
n_modifications = len(user_params['modif_dict'])
tot_number_mod = n_modifications**n_reactions
print(f"Total number of possible modifications: {tot_number_mod}")
trainingset = user_params['n_instances']/tot_number_mod*100
print(f"Training set size: {user_params['n_instances'] } ({trainingset:.2f}% of the total)")
```

```
Total number of possible modifications: 6561
Training set size: 96 (1.46% of the total)
```

## Generating initial designs by running ART¶

Define a dictionary that contains the settings that ART will use to find the recommended designs:

In [8]:

```
art_params = {
    'input_var': user_params['reactions'],                    # input variables, i.e. features
    'num_recommendations': user_params['n_instances'] - 1,    # one of them will be wild type
    'initial_cycle': True,                                    # Set this to True for initial designs recommendations
    'seed': 10,                                               # seed for number random generator
    'output_directory': '../data/art_output/initial_designs'  # directory to store this output
}
```

As current version of ART works only with continuous variables, we will first find recommended designs in the interval [0, 1] and then transform each of those to one of numerical categories defined above {0, 1, 2} by using the floor function

$$f(x) = 3\lfloor x \rfloor$$

With the configuration stored in art\_params, we now run ART:

In [9]:

```
art = RecommendationEngine(**art_params)
df = art.recommendations.copy()
df.tail()
```

Out[9]:

|  | ACCOAC | MDH | PTAr | CS | ACACT1r | PPC | PPCK | PFL |
| --- | --- | --- | --- | --- | --- | --- | --- | --- |
| 90 | 0.834820 | 0.310928 | 0.017741 | 0.686528 | 0.038425 | 0.702041 | 0.909326 | 0.314650 |
| 91 | 0.253443 | 0.144406 | 0.797025 | 0.866835 | 0.196778 | 0.915534 | 0.227534 | 0.997582 |
| 92 | 0.448214 | 0.982897 | 0.229292 | 0.020562 | 0.236674 | 0.920214 | 0.671678 | 0.159832 |
| 93 | 0.058396 | 0.672483 | 0.191037 | 0.602885 | 0.087692 | 0.708920 | 0.656125 | 0.545103 |
| 94 | 0.012306 | 0.396266 | 0.981174 | 0.236980 | 0.613428 | 0.391463 | 0.127033 | 0.926296 |

And transform the initial designs to categories (0, 1 or 2) by using the floor function:

In [10]:

```
df = np.floor(3 * df)
df.tail()
```

Out[10]:

|  | ACCOAC | MDH | PTAr | CS | ACACT1r | PPC | PPCK | PFL |
| --- | --- | --- | --- | --- | --- | --- | --- | --- |
| 90 | 2.0 | 0.0 | 0.0 | 2.0 | 0.0 | 2.0 | 2.0 | 0.0 |
| 91 | 0.0 | 0.0 | 2.0 | 2.0 | 0.0 | 2.0 | 0.0 | 2.0 |
| 92 | 1.0 | 2.0 | 0.0 | 0.0 | 0.0 | 2.0 | 2.0 | 0.0 |
| 93 | 0.0 | 2.0 | 0.0 | 1.0 | 0.0 | 2.0 | 1.0 | 1.0 |
| 94 | 0.0 | 1.0 | 2.0 | 0.0 | 1.0 | 1.0 | 0.0 | 2.0 |

### Adding the wild type strain¶

We include as last design the wild type strain, since we will use this list to find isoprenol production:

In [11]:

```
df.loc[user_params['n_instances']-1] = [user_params['modif_dict']['NoMod'] for i in range(n_reactions)] 
df = df.astype(int)
df.tail()
```

Out[11]:

|  | ACCOAC | MDH | PTAr | CS | ACACT1r | PPC | PPCK | PFL |
| --- | --- | --- | --- | --- | --- | --- | --- | --- |
| 91 | 0 | 0 | 2 | 2 | 0 | 2 | 0 | 2 |
| 92 | 1 | 2 | 0 | 0 | 0 | 2 | 2 | 0 |
| 93 | 0 | 2 | 0 | 1 | 0 | 2 | 1 | 1 |
| 94 | 0 | 1 | 2 | 0 | 1 | 1 | 0 | 2 |
| 95 | 1 | 1 | 1 | 1 | 1 | 1 | 1 | 1 |

### Checking the distribution of initial designs¶

The distribution of initial desings is approximately the same for each category (i.e. modification):

In [12]:

```
plot_distribution_of_designs(df)
```

## Saving the generated designs¶

First we need ot create the line names for all designs:

In [13]:

```
df.insert(loc=0, column='Line Name', value=['Strain ' + str(i) for i in range(1,user_params['n_instances'])] + ['WT'])
df.tail()
```

Out[13]:

|  | Line Name | ACCOAC | MDH | PTAr | CS | ACACT1r | PPC | PPCK | PFL |
| --- | --- | --- | --- | --- | --- | --- | --- | --- | --- |
| 91 | Strain 92 | 0 | 0 | 2 | 2 | 0 | 2 | 0 | 2 |
| 92 | Strain 93 | 1 | 2 | 0 | 0 | 0 | 2 | 2 | 0 |
| 93 | Strain 94 | 0 | 2 | 0 | 1 | 0 | 2 | 1 | 1 |
| 94 | Strain 95 | 0 | 1 | 2 | 0 | 1 | 1 | 0 | 2 |
| 95 | WT | 1 | 1 | 1 | 1 | 1 | 1 | 1 | 1 |

Add here the appropriate PI name and email:

In [14]:

```
PI       = "Hector Garcia Martin"  # Change to your PI!!!
PI_email = "hgmartin@lbl.gov"      # Cahnge to your PI's email!!!!
```

And then we proceed to generate the ICE file:

In [15]:

```
header = 'Principal Investigator*, Principal Investigator Email, \
            Funding Source, Intellectual Property, BioSafety Level*, Name*, Alias, \
            Keywords, Summary*, Notes, References, Links, Status*, Creator*, Creator Email*, Host, \
            Genotype or Phenotype, Selection Markers*, Sequence Trace File(s), Sequence File, \
            Attachment File'
append_pi   = PI + ', ' + PI_email + ', , , 1'
append_desc = ', , , Complete, ' + PI + ', ' + PI_email + ', , , , , , '

with open(user_params['designs_ice_file'], 'w') as fh:
    
    fh.write(f'{header}\n')
    
    for i in range(len(df)) :
        if df.loc[i, "Line Name"] == 'WT':
            strain_name = 'WT'
            strain_description = 'Wild type E. coli'
        else:
            strain_name = df.loc[i, "Line Name"]
            strain_description = '_'.join([f'{reaction}_{df.loc[i, reaction]}' for reaction in user_params['reactions']])
        
        fh.write(f'{append_pi},{strain_name}, , ,{strain_description},{append_desc}\n')
```

In [ ]:

```

```
