# Supplementary material for: Multiomics Data Collection, Visualization, and Utilization for Guiding Metabolic Engineering
Source: Front Bioeng Biotechnol. 2021 Feb 9;9:612893. doi: 10.3389/fbioe.2021.612893 (PMC7902046; doi:10.3389/fbioe.2021.612893)
Supplement: Supplementary file 11 [file Data_Sheet_2.ZIP › NotebookC.html]

C\_Generate\_isoprenol\_concentrations


# Notebook C: Calculation of isoprenol concentrations for different strain designs¶

This notebook takes the initial designs previously suggested by ART and uses OMG to create the corresponding final isoprenol concentrations. These concentrations, along with the designs will be later used by ART to make predictions and recommend new designs.

Tested using **biodesign\_3.7** kernel on jprime.lbl.gov server. It requires the cplex library for running the MOMA optimization.

## Inputs and outputs¶

#### Required files to run this notebook:¶

- A modified E. coli model with the isoprenol pathway added to it (`iJO1366_MVA.json` file in the `../data/models` directory)
- A set of designs (e.g. `../data/ice_mo_strains.csv` exported from **ICE**) containing the details of which reactions are either:
  - (0) eliminated
  - (1) included
  - (2) doubled the flux

#### Files generated by running this notebook:¶

- `EDD_experiment_description_file_BE_designs.csv`
- `EDD_isoprenol_production.csv`

The files are stored in the user defined directory.

## Setup¶

Clone the git repository with the `OMG` library:

`git clone https://github.com/JBEI/OMG.git`

or pull the latest version.

Importing needed libraries:

In [1]:

```
import sys
import os

sys.path.insert(1, '../../OMG')
sys.path.append('../')

import cobra
import pandas as pd
import omg 
from plot_multiomics import *
from tqdm import tqdm
```

## User parameters¶

In [2]:

```
user_params = {
    'host': 'ecoli', # ecoli or ropacus
    'modelfile': '../data/models/iJO1366_MVA.json',
    'cerevisiae_modelfile': '../data/models/iMM904.json',
    'timestart': 0.0,
    'timestop': 8.0,
    'numtimepoints': 9,
    'designsfile': 'ice_mo_strains.csv',
    'designsfilepath': '../data/',
    'mapping_file': '../mapping/inchikey_to_cid.txt',
    'output_file_path': '../data/omg_output',
    'edd_omics_file_path': '../data/omg_output/edd/',
    'numreactions': 8,
    'numinstances': 96,
    'ext_metabolites': {
        'glc__D_e': 22.203,
        'nh4_e': 18.695,
        'pi_e': 69.454,
        'so4_e': 2.0,
        'mg2_e': 2.0,
        'k_e': 21.883,
        'na1_e': 103.7,
        'cl_e': 27.25,
        'isoprenol_e': 0.0,
        'ac_e': 0.0,
        'for_e': 0.0,
        'lac__D_e': 0.0,
        'etoh_e': 0.0
    },
    'initial_OD': 0.01,
    'BIOMASS_REACTION_ID': 'BIOMASS_Ec_iJO1366_core_53p95M'
}
```

## Using the OMG library functions for creating synthetic multiomics data¶

### 1) Getting and preparing the metabolic model¶

First we obtain the metabolic model:

In [3]:

```
file_name = user_params['modelfile']
model = cobra.io.load_json_model(file_name)
```

We now add minimum flux constraints for production of isoprenol and formate, and we limit oxygen intake:

In [4]:

```
iso = 'EX_isoprenol_e'
iso_cons = model.problem.Constraint(model.reactions.EX_isoprenol_e.flux_expression,lb = 0.20)
model.add_cons_vars(iso_cons)
for_cons = model.problem.Constraint(model.reactions.EX_for_e.flux_expression,lb = 0.10)
model.add_cons_vars(for_cons)
o2_cons = model.problem.Constraint(model.reactions.EX_o2_e.flux_expression,lb = -8.0)
model.add_cons_vars(o2_cons)
```

And then we constrain several central carbon metabolism fluxes to more realistic upper and lower bounds:

In [5]:

```
CC_rxn_names = ['ACCOAC','MDH','PTAr','CS','ACACT1r','PPC','PPCK','PFL']
for reaction in CC_rxn_names:
    reaction_constraint = model.problem.Constraint(model.reactions.get_by_id(reaction).flux_expression,lb = -1.0,ub =  1.0)
    model.add_cons_vars(reaction_constraint)
```

We also create a similar model with a higher production of isoprenol, which we will use with MOMA to simulate bioengineered strains:

In [6]:

```
modelHI = model.copy()
iso_cons = modelHI.problem.Constraint(modelHI.reactions.EX_isoprenol_e.flux_expression,lb = 0.25)
modelHI.add_cons_vars(iso_cons)
```

### 2) Obtaining times series for the wild type¶

First create the time grid for simulation:

In [7]:

```
t0 = user_params['timestart']
tf = user_params['timestop']
points = user_params['numtimepoints']
tspan, delt = np.linspace(t0, tf, points, dtype='float64', retstep=True)

grid = (tspan, delt)
```

We then use this model to obtain the times series for fluxes, OD and external metabolites:

In [8]:

```
solution_TS, model_TS, cell, Emets, Erxn2Emet = \
    omg.get_flux_time_series(model, user_params['ext_metabolites'], grid, user_params)
```

```
0.0 optimal 0.5363612610171437
1.0 optimal 0.5363612610171437
2.0 optimal 0.5363612610171437
3.0 optimal 0.5363612610171437
4.0 optimal 0.5363612610171437
5.0 optimal 0.5363612610171437
6.0 optimal 0.5363612610171437
7.0 optimal 0.5363612610171437
8.0 optimal 0.5363612610171437
```

We perform the same calculation for the model with higher isoprenol production that we created above:

In [9]:

```
solutionHI_TS, modelHI_TS, cellHI, EmetsHI, Erxn2EmetHI = \
    omg.get_flux_time_series(modelHI, user_params['ext_metabolites'], grid, user_params)
```

```
0.0 optimal 0.5352266385352652
1.0 optimal 0.5352266385352652
2.0 optimal 0.5352266385352652
3.0 optimal 0.5352266385352652
4.0 optimal 0.5352266385352652
5.0 optimal 0.5352266385352652
6.0 optimal 0.5352266385352652
7.0 optimal 0.5352266385352652
8.0 optimal 0.5352266385352652
```

### 3) Getting bioengineered flux profiles through MOMA¶

First obtain the file from ICE with suggested designs (i.e. reactions kos and overexpressions):

In [10]:

```
designs_df = pd.read_csv(f'{user_params["designsfilepath"]}/{user_params["designsfile"]}',
                        usecols=['Part ID', 'Name', 'Summary'])
designs_df.columns = ['Part ID','Line Name','Line Description']
designs_df2 = designs_df.copy()  # make copy for creating EDD experiment description file later
designs_df[:2]
```

Out[10]:

|  | Part ID | Line Name | Line Description |
| --- | --- | --- | --- |
| 0 | ABFPUB\_000215 | Strain 1 | ACCOAC\_1.0\_MDH\_1.0\_PTAr\_2.0\_CS\_0.0\_ACACT1r\_2.0... |
| 1 | ABFPUB\_000216 | Strain 2 | ACCOAC\_1.0\_MDH\_2.0\_PTAr\_2.0\_CS\_2.0\_ACACT1r\_2.0... |

#### Storing information from ICE line description into a dataframe.¶

In order to work with the ICE line descriptions we need to change it from its string format into numerical format into a dataframe (see below).

First, let's add columns for each reaction:

In [11]:

```
reactions = designs_df['Line Description'][0].split('_')[::2]
for rxn in reactions:
    designs_df[rxn] = None
```

And then assign values for each reaction and line

In [12]:

```
for i in range(len(designs_df)):
    if designs_df['Line Name'][i]=='WT':
        designs_df.loc[i][reactions] = [1 for r in range(len(reactions))]
    else:
        values = designs_df.loc[i]['Line Description'].split('_')[1::2]
        designs_df.loc[i][reactions] = [float(value) for value in values]

designs_df = designs_df.drop(columns=['Line Description','Part ID'])
```

The final dataframe involves the line name and numerical multiplier that we will use to simulate the bioengineered strains.
Each design (line) involves the modification of up to 8 fluxes (1 -> keep the same; 2-> double flux, 0-> knock reaction out):

In [13]:

```
designs_df.tail()
```

Out[13]:

|  | Line Name | ACCOAC | MDH | PTAr | CS | ACACT1r | PPC | PPCK | PFL |
| --- | --- | --- | --- | --- | --- | --- | --- | --- | --- |
| 91 | Strain 92 | 0 | 0 | 2 | 2 | 0 | 2 | 0 | 2 |
| 92 | Strain 93 | 1 | 2 | 0 | 0 | 0 | 2 | 2 | 0 |
| 93 | Strain 94 | 0 | 2 | 0 | 1 | 0 | 2 | 1 | 1 |
| 94 | Strain 95 | 0 | 1 | 2 | 0 | 1 | 1 | 0 | 2 |
| 95 | WT | 1 | 1 | 1 | 1 | 1 | 1 | 1 | 1 |

#### Creating time series of flux profiles for each bioengineered strain¶

We then use MOMA to calculate flux profiles at each time point for the bioengineered strains as indicated by the designs in this data frame (takes around 1 min per design). Instead of using the solution time series corresponding to the initial model, we use the solution time series corresponding to the higher production. The reason is that, otherwise, we would never see an increase in isoprenol production, since MOMA minimizes the changes in flux by design. Our goal here is just to create varied flux profiles that ART can learn from.

This is a **long calculation** (~1.5 hrs):

In [14]:

```
%%time
solutionsMOMA_TS = {}
cols = ['Line Name']
cols.extend(reactions)

if user_params['numinstances'] not in [None, 0]:
    num_strains = user_params['numinstances']
else:
    num_strains = designs_df.shape[0]
    
for i in tqdm(range(num_strains)):      # Added counter bar here
    design = designs_df[cols].loc[i]
    if design['Line Name']=='WT':
        solutionsMOMA_TS[i] = omg.getBEFluxes(model_TS, design, solution_TS, grid)
    else:
        solutionsMOMA_TS[i] = omg.getBEFluxes(model_TS, design, solutionHI_TS, grid)
```

```
100%|██████████| 96/96 [1:30:58<00:00, 56.86s/it]
```

```
CPU times: user 1h 30min 8s, sys: 39.6 s, total: 1h 30min 47s
Wall time: 1h 30min 58s
```

```

```

As a sanity check, we can verify that the knocked out fluxes are zero:

In [15]:

```
i = 0
print(designs_df.loc[i,:], '\n')
for rxn in ['CS','PPC','PPCK','PFL']:
    print(f'{rxn}: {solutionsMOMA_TS[i][5].fluxes[rxn]}')
```

```
Line Name    Strain 1
ACCOAC              1
MDH                 1
PTAr                2
CS                  0
ACACT1r             2
PPC                 0
PPCK                0
PFL                 0
Name: 0, dtype: object 

CS: 0.0
PPC: 0.0
PPCK: 0.0
PFL: 0.0
```

### 4) Producing the external metabolite concentrations for each bioengineered strain¶

Here we use the `integrate_fluxes` function in OMG to produce the external metabolite concentrations which are the consequence of the calculated fluxes:

In [16]:

```
cellsEmetsBE = {}
for i in range(num_strains):
    cell, Emets = omg.integrate_fluxes(solutionsMOMA_TS[i], model_TS, user_params['ext_metabolites'], grid, user_params)
    cellsEmetsBE[i] = (cell, Emets)
```

We can check we obtain the same result with this function for the wild type as we did before in notebook A:

In [17]:

```
cellWT, EmetsWT = omg.integrate_fluxes(solution_TS, model_TS, user_params['ext_metabolites'], grid, user_params)
```

In [18]:

```
EmetsWT
```

Out[18]:

|  | glc\_\_D\_e | nh4\_e | pi\_e | so4\_e | mg2\_e | k\_e | na1\_e | cl\_e | isoprenol\_e | ac\_e | for\_e | lac\_\_D\_e | etoh\_e |
| --- | --- | --- | --- | --- | --- | --- | --- | --- | --- | --- | --- | --- | --- |
| 0.0 | 22.0707 | 18.6183 | 69.4472 | 1.99821 | 1.99994 | 21.8816 | 103.7 | 27.25 | 0.00264663 | 0.027405 | 0.00132331 | 0.102538 | 0 |
| 1.0 | 21.8444 | 18.4873 | 69.4354 | 1.99515 | 1.99983 | 21.8792 | 103.7 | 27.2499 | 0.00717176 | 0.0742613 | 0.00358588 | 0.277855 | 0 |
| 2.0 | 21.4576 | 18.2632 | 69.4154 | 1.98992 | 1.99965 | 21.8752 | 103.7 | 27.2498 | 0.0149087 | 0.154375 | 0.00745436 | 0.577608 | 0 |
| 3.0 | 20.7961 | 17.88 | 69.3812 | 1.98097 | 1.99935 | 21.8683 | 103.7 | 27.2496 | 0.0281372 | 0.291351 | 0.0140686 | 1.09012 | 0 |
| 4.0 | 19.6653 | 17.2248 | 69.3227 | 1.96567 | 1.99882 | 21.8564 | 103.7 | 27.2493 | 0.0507548 | 0.52555 | 0.0253774 | 1.96639 | 0 |
| 5.0 | 17.7317 | 16.1047 | 69.2227 | 1.93951 | 1.99792 | 21.8362 | 103.7 | 27.2488 | 0.0894259 | 0.925977 | 0.044713 | 3.46462 | 0 |
| 6.0 | 14.4258 | 14.1895 | 69.0516 | 1.89479 | 1.99638 | 21.8016 | 103.7 | 27.2478 | 0.155545 | 1.61062 | 0.0777724 | 6.02626 | 0 |
| 7.0 | 8.77335 | 10.915 | 68.7592 | 1.81833 | 1.99375 | 21.7424 | 103.7 | 27.2463 | 0.268593 | 2.7812 | 0.134297 | 10.4061 | 0 |
| 8.0 | 0 | 5.31631 | 68.2591 | 1.68759 | 1.98925 | 21.6412 | 103.7 | 27.2436 | 0.46188 | 4.78262 | 0.23094 | 17.8946 | 0 |
| 9.0 | 0 | 5.31631 | 68.2591 | 1.68759 | 1.98925 | 21.6412 | 103.7 | 27.2436 | 0.46188 | 4.78262 | 0.23094 | 17.8946 | 0 |

In [19]:

```
plot_DO_extmets(cellWT, EmetsWT[['glc__D_e','isoprenol_e','ac_e','for_e','lac__D_e','etoh_e']])
```

And compare these growth and production profiles with any other bioengineered strain:

In [20]:

```
i = 2
cellBE, EmetsBE = cellsEmetsBE[i]
plot_DO_extmets(cellBE, EmetsBE[['glc__D_e','isoprenol_e','ac_e','for_e','lac__D_e','etoh_e']])
```

In [21]:

```
EmetsBE
```

Out[21]:

|  | glc\_\_D\_e | nh4\_e | pi\_e | so4\_e | mg2\_e | k\_e | na1\_e | cl\_e | isoprenol\_e | ac\_e | for\_e | lac\_\_D\_e | etoh\_e |
| --- | --- | --- | --- | --- | --- | --- | --- | --- | --- | --- | --- | --- | --- |
| 0.0 | 22.0752 | 18.6209 | 69.4481 | 1.99814 | 1.99995 | 21.8818 | 103.7 | 27.25 | 0.00305532 | 0.0274419 | 0.00127792 | 0.0961986 | 0 |
| 1.0 | 21.8703 | 18.5022 | 69.4387 | 1.99517 | 1.99986 | 21.8799 | 103.7 | 27.2499 | 0.00795333 | 0.0714341 | 0.00332657 | 0.250415 | 0 |
| 2.0 | 21.5419 | 18.3118 | 69.4236 | 1.99039 | 1.99973 | 21.8769 | 103.7 | 27.2498 | 0.0158052 | 0.141958 | 0.00661077 | 0.497641 | 0 |
| 3.0 | 21.0154 | 18.0067 | 69.3994 | 1.98274 | 1.99951 | 21.8721 | 103.7 | 27.2497 | 0.0283926 | 0.255016 | 0.0118757 | 0.893971 | 0 |
| 4.0 | 20.1714 | 17.5175 | 69.3606 | 1.97048 | 1.99917 | 21.8643 | 103.7 | 27.2495 | 0.048572 | 0.436261 | 0.0203159 | 1.52933 | 0 |
| 5.0 | 18.8183 | 16.7333 | 69.2984 | 1.95081 | 1.99861 | 21.8518 | 103.7 | 27.2492 | 0.0809214 | 0.726815 | 0.0338466 | 2.54788 | 0 |
| 6.0 | 16.6492 | 15.4761 | 69.1986 | 1.91929 | 1.99773 | 21.8318 | 103.7 | 27.2486 | 0.132781 | 1.1926 | 0.0555376 | 4.18072 | 0 |
| 7.0 | 13.1719 | 13.4607 | 69.0388 | 1.86876 | 1.9963 | 21.7998 | 103.7 | 27.2478 | 0.215919 | 1.93931 | 0.0903106 | 6.79835 | 0 |
| 8.0 | 7.59744 | 10.2297 | 68.7824 | 1.78775 | 1.99402 | 21.7485 | 103.7 | 27.2464 | 0.349196 | 3.13637 | 0.146056 | 10.9947 | 0 |
| 9.0 | 7.59744 | 10.2297 | 68.7824 | 1.78775 | 1.99402 | 21.7485 | 103.7 | 27.2464 | 0.349196 | 3.13637 | 0.146056 | 10.9947 | 0 |

### 5) Creating a file with isoprenol concentrations for EDD import and training ART¶

Firstly, let's collect all isoprenol production values in a single list:

In [22]:

```
production = []
for i in range(user_params['numinstances']):
    cell, Emets = cellsEmetsBE[i]
    production.append(Emets.loc[user_params['numtimepoints'],'isoprenol_e'])
```

Then, let's create a new data frame and append the production values for each strain/line:

In [23]:

```
production_df = designs_df.copy()
production_df['Isoprenol'] = pd.Series(production)
production_df.loc[0:2,:]
```

Out[23]:

|  | Line Name | ACCOAC | MDH | PTAr | CS | ACACT1r | PPC | PPCK | PFL | Isoprenol |
| --- | --- | --- | --- | --- | --- | --- | --- | --- | --- | --- |
| 0 | Strain 1 | 1 | 1 | 2 | 0 | 2 | 0 | 0 | 0 | 0.000000 |
| 1 | Strain 2 | 1 | 2 | 2 | 2 | 2 | 1 | 1 | 0 | 0.552101 |
| 2 | Strain 3 | 1 | 0 | 0 | 2 | 1 | 1 | 2 | 0 | 0.349196 |

The maximum production is higher than for the original (WT) strain (0.462):

In [24]:

```
np.max(production_df['Isoprenol'])
```

Out[24]:

```
0.5726483723065023
```

#### Reformat for export as EDD input file¶

Remove not needed columns:

In [25]:

```
production_edd_df = production_df.drop(columns=reactions).copy()
```

Rename isoprenol column:

In [26]:

```
isoprenol_cid = 'CID:12988'
production_edd_df = production_edd_df.rename(columns={'Isoprenol': isoprenol_cid})
```

Pivot the dataframe for EDD format:

In [27]:

```
production_edd_df = production_edd_df.set_index('Line Name').stack().reset_index()
production_edd_df.columns = ['Line Name', 'Measurement Type', 'Value']
```

Add Time and Units columns:

In [28]:

```
production_edd_df['Time'] = 9.0
production_edd_df['Units'] = 'mM'
production_edd_df.head()
```

Out[28]:

|  | Line Name | Measurement Type | Value | Time | Units |
| --- | --- | --- | --- | --- | --- |
| 0 | Strain 1 | CID:12988 | 0.000000 | 9.0 | mM |
| 1 | Strain 2 | CID:12988 | 0.552101 | 9.0 | mM |
| 2 | Strain 3 | CID:12988 | 0.349196 | 9.0 | mM |
| 3 | Strain 4 | CID:12988 | 0.551849 | 9.0 | mM |
| 4 | Strain 5 | CID:12988 | 0.080117 | 9.0 | mM |

Save the dataframe as csv:

In [29]:

```
production_file_name = f'{user_params["edd_omics_file_path"]}/EDD_isoprenol_production.csv'
production_edd_df.to_csv(production_file_name, index=False)
```

#### Create experiment description file for EDD¶

We then create the `EDD_experiment_description_file_BE_designs.csv` file for the import of data into EDD:

In [30]:

```
experiment_description_file_name = f'{user_params["edd_omics_file_path"]}/EDD_experiment_description_file_BE_designs.csv'

with open(experiment_description_file_name, 'w') as fh:
    fh.write('Part ID, Line Name, Line Description, Media, Shaking Speed, Starting OD, Culture Volume, Flask Volume, Growth Temperature, Replicate Count\n')
    for i in range(len(designs_df2)):
        fh.write(f"{designs_df2.loc[i]['Part ID']}, \
                 {designs_df2.loc[i]['Line Name']}, \
                 {designs_df2.loc[i]['Line Description']}, \
                 M9, 1, 0.1, 50, 200, 30, 1\n")
```

In [ ]:

```

```
