# Supplementary material for: Multiomics Data Collection, Visualization, and Utilization for Guiding Metabolic Engineering
Source: Front Bioeng Biotechnol. 2021 Feb 9;9:612893. doi: 10.3389/fbioe.2021.612893 (PMC7902046; doi:10.3389/fbioe.2021.612893)
Supplement: Supplementary file 11 [file Data_Sheet_2.ZIP › NotebookE.html]

E\_Generate\_omics\_data\_bestrec


# Notebook E: Calculation of isoprenol concentrations for the recommendations generated using ART¶

This notebook takes the machine learning predictions from the previous notebook and compares them with the ground truth provided by OMG.

Tested using **biodesign\_3.7** kernel on jprime.lbl.gov. It requires the cplex library for running the MOMA optimization.

## Inputs and Outputs¶

#### Required files to run this notebook:¶

- A modified E. coli model with the isoprenol pathway added to it (`iJO1366_MVA.json` file in the `../data/models` directory)
- A set of recommended designs provided by ART (`../data/art_output/ARTrecommendations.csv`)

#### Files generated by running this notebook:¶

- A file containing all the recommendations with their associated actual levels of production (`../data/ART_recommendations_with_production.csv`).

- Plot of predicted vs observed levels of production for all recommendations (`../data/ART_prediction_vs_actual_recommendation.png`).

- For EDD import:
  - `EDD_experiment_description_file_BT.csv`
  - `EDD_OD_BT.csv`
  - `EDD_external_metabolites_BT.csv`
  - `EDD_transcriptomics_BT.csv`
  - `EDD_proteomics_BTSM.csv`
  - `EDD_metabolomics_BTSM.csv`
  - `EDD_fluxomics_BT.csv`

## Setup¶

Clone the git repository with the `OMG` library:

`git clone https://github.com/JBEI/OMG.git`

or pull the latest version.

In [1]:

```
import sys
import os

sys.path.insert(1, '../../OMG')
sys.path.append('../')

import cobra
import pandas as pd
import omg 
from plot_multiomics import *
from tqdm import tqdm
```

## User parameters¶

In [2]:

```
user_params = {
    'host': 'ecoli', # ecoli or ropacus
    'modelfile': '../data/models/iJO1366_MVA.json',
    'cerevisiae_modelfile': '../data/models/iMM904.json', 
    'timestart': 0.0,
    'timestop': 8.0,
    'numtimepoints': 9,
    'designsfile': 'ARTrecommendations.csv',
    'designsfilepath': '../data/art_output',
    'mapping_file': '../mapping/inchikey_to_cid.txt',
    'output_file_path': 'data/omg_output',
    'edd_omics_file_path': '../data/omg_output/edd/',
    'numreactions': 8,
    'numinstances': 10,
    'ext_metabolites': {
        'glc__D_e': 22.203,
        'nh4_e': 18.695,
        'pi_e': 69.454,
        'so4_e': 2.0,
        'mg2_e': 2.0,
        'k_e': 21.883,
        'na1_e': 103.7,
        'cl_e': 27.25,
        'isoprenol_e': 0.0,
        'ac_e': 0.0,
        'for_e': 0.0,
        'lac__D_e': 0.0,
        'etoh_e': 0.0
    },
    'initial_OD': 0.01,
    'BIOMASS_REACTION_ID': 'BIOMASS_Ec_iJO1366_core_53p95M'
}
```

## Using the OMG library functions for creating synthetic multiomics data for recommended designs¶

### 1) Getting and preparing the metabolic model¶

We proceed in the same way as in Notebook A

First we obtain the metabolic model:

In [3]:

```
file_name = user_params['modelfile']
model = cobra.io.load_json_model(file_name)
```

We now add minimum flux constraints for production of isoprenol and formate, and we limit oxygen intake:

In [4]:

```
iso = 'EX_isoprenol_e'
iso_cons = model.problem.Constraint(model.reactions.EX_isoprenol_e.flux_expression,lb = 0.20)
model.add_cons_vars(iso_cons)
for_cons = model.problem.Constraint(model.reactions.EX_for_e.flux_expression,lb = 0.10)
model.add_cons_vars(for_cons)
o2_cons = model.problem.Constraint(model.reactions.EX_o2_e.flux_expression,lb = -8.0)
model.add_cons_vars(o2_cons)
```

And then we constrain several central carbon metabolism fluxes to more realistic upper and lower bounds:

In [5]:

```
CC_rxn_names = ['ACCOAC','MDH','PTAr','CS','ACACT1r','PPC','PPCK','PFL']
for reaction in CC_rxn_names:
    reaction_constraint = model.problem.Constraint(model.reactions.get_by_id(reaction).flux_expression,lb = -1.0,ub =  1.0)
    model.add_cons_vars(reaction_constraint)
```

We also create a similar model with a higher production of isoprenol, which we will use with MOMA to simulate bioengineered strains:

In [6]:

```
modelHI = model.copy()
iso_cons = modelHI.problem.Constraint(modelHI.reactions.EX_isoprenol_e.flux_expression,lb = 0.25)
modelHI.add_cons_vars(iso_cons)
```

### 2) Obtaining times series for the wild type¶

We will need this to create time series for bioenginered strains. First, we create first time grid for simulation:

In [7]:

```
t0 = user_params['timestart']
tf = user_params['timestop']
points = user_params['numtimepoints']
tspan, delt = np.linspace(t0, tf, points, dtype='float64', retstep=True)

grid = (tspan, delt)
```

We then use this model to obtain the times series for fluxes, OD and external metabolites:

In [8]:

```
solution_TS, model_TS, cell, Emets, Erxn2Emet = \
    omg.get_flux_time_series(model, user_params['ext_metabolites'], grid, user_params)
```

```
0.0 optimal 0.5363612610171437
1.0 optimal 0.5363612610171437
2.0 optimal 0.5363612610171437
3.0 optimal 0.5363612610171437
4.0 optimal 0.5363612610171437
5.0 optimal 0.5363612610171437
6.0 optimal 0.5363612610171437
7.0 optimal 0.5363612610171437
8.0 optimal 0.5363612610171437
```

We perform the same calculation for the model with higher isoprenol production that we created above:

In [9]:

```
solutionHI_TS, modelHI_TS, cellHI, EmetsHI, Erxn2EmetHI = \
    omg.get_flux_time_series(modelHI, user_params['ext_metabolites'], grid, user_params)
```

```
0.0 optimal 0.5352266385352652
1.0 optimal 0.5352266385352652
2.0 optimal 0.5352266385352652
3.0 optimal 0.5352266385352652
4.0 optimal 0.5352266385352652
5.0 optimal 0.5352266385352652
6.0 optimal 0.5352266385352652
7.0 optimal 0.5352266385352652
8.0 optimal 0.5352266385352652
```

### 3) Getting bioengineered flux profiles through MOMA¶

Read the file from ART with suggested designs (i.e. reactions kos and overexpressions):

In [10]:

```
rec_df = pd.read_csv(f'{user_params["designsfilepath"]}/{user_params["designsfile"]}')
rec_df.head()
```

Out[10]:

|  | Line Name | ACCOAC | MDH | PTAr | CS | ACACT1r | PPC | PPCK | PFL | Mean predicted Isoprenol [mM] | SD Isoprenol [mM] |
| --- | --- | --- | --- | --- | --- | --- | --- | --- | --- | --- | --- |
| 0 | Strain 97 | 2.0 | 2.0 | 1.0 | 2.0 | 2.0 | 2.0 | 0.0 | 1.0 | 0.573584 | 0.019785 |
| 1 | Strain 98 | 2.0 | 2.0 | 2.0 | 2.0 | 2.0 | 2.0 | 0.0 | 1.0 | 0.572193 | 0.019752 |
| 2 | Strain 99 | 1.0 | 2.0 | 1.0 | 2.0 | 2.0 | 2.0 | 0.0 | 1.0 | 0.572167 | 0.019752 |
| 3 | Strain 100 | 2.0 | 1.0 | 1.0 | 2.0 | 2.0 | 2.0 | 0.0 | 1.0 | 0.572039 | 0.019787 |
| 4 | Strain 101 | 2.0 | 2.0 | 1.0 | 2.0 | 2.0 | 1.0 | 0.0 | 1.0 | 0.571967 | 0.019780 |

We then use MOMA to calculate flux profiles at each time point for the recommended strains. Instead of using the solution time series corresponding to the initial model, we use the solution time series corresponding to the higher production. The reason is that, otherwise, we would never see an increase in isoprenol production, since MOMA minimizes the changes in flux by design. Remember that our goal here is just to create realistic flux profiles that can be used to showcase ART. This approach is good enough for that purpose:

In [11]:

```
%%time
solutionsMOMA_TS = {}
cols = rec_df.columns[:-2]

if user_params['numinstances'] not in [None, 0]:
    num_strains = user_params['numinstances']
else:
    num_strains = rec_df.shape[0]

for i in tqdm(range(num_strains)):
    design = rec_df[cols].loc[i]
    if design['Line Name']=='WT':
        solutionsMOMA_TS[i] = omg.getBEFluxes(model_TS, design, solution_TS, grid)
    else:
        solutionsMOMA_TS[i] = omg.getBEFluxes(model_TS, design, solutionHI_TS, grid)
```

```
100%|██████████| 10/10 [09:58<00:00, 59.84s/it]
```

```
CPU times: user 9min 53s, sys: 3.82 s, total: 9min 57s
Wall time: 9min 58s
```

```

```

### 4) Producing the external metabolite concentrations for each recommendation¶

Here we use the `integrate_fluxes` function to produce the external metabolite concentrations compatible with the calculated fluxes:

In [12]:

```
cellsEmetsBE = {}
for i in range(num_strains):
    cell, Emets = omg.integrate_fluxes(solutionsMOMA_TS[i], model_TS, user_params['ext_metabolites'], grid, user_params)
    cellsEmetsBE[i] = (cell, Emets)
```

We can visualize the obtained concentrations for a recommendation:

In [13]:

```
i = 2
cellBE, EmetsBE = cellsEmetsBE[i]
plot_DO_extmets(cellBE, EmetsBE[['glc__D_e','isoprenol_e','ac_e','for_e','lac__D_e','etoh_e']])
```

In [14]:

```
EmetsBE
```

Out[14]:

|  | glc\_\_D\_e | nh4\_e | pi\_e | so4\_e | mg2\_e | k\_e | na1\_e | cl\_e | isoprenol\_e | ac\_e | for\_e | lac\_\_D\_e | etoh\_e |
| --- | --- | --- | --- | --- | --- | --- | --- | --- | --- | --- | --- | --- | --- |
| 0.0 | 22.0708 | 18.6185 | 69.4472 | 1.99821 | 1.99994 | 21.8816 | 103.7 | 27.25 | 0.00330624 | 0.0287831 | 0.0013225 | 0.100218 | 0 |
| 1.0 | 21.8449 | 18.488 | 69.4355 | 1.99517 | 1.99983 | 21.8793 | 103.7 | 27.2499 | 0.00895276 | 0.0779399 | 0.0035811 | 0.271374 | 0 |
| 2.0 | 21.4592 | 18.265 | 69.4156 | 1.98996 | 1.99965 | 21.8752 | 103.7 | 27.2498 | 0.0185961 | 0.161892 | 0.00743843 | 0.563679 | 0 |
| 3.0 | 20.8004 | 17.8842 | 69.3816 | 1.98107 | 1.99935 | 21.8683 | 103.7 | 27.2496 | 0.0350653 | 0.305267 | 0.0140261 | 1.06289 | 0 |
| 4.0 | 19.6753 | 17.2338 | 69.3235 | 1.96588 | 1.99883 | 21.8566 | 103.7 | 27.2493 | 0.0631919 | 0.550129 | 0.0252768 | 1.91546 | 0 |
| 5.0 | 17.7539 | 16.123 | 69.2243 | 1.93994 | 1.99793 | 21.8365 | 103.7 | 27.2488 | 0.111228 | 0.968313 | 0.0444911 | 3.3715 | 0 |
| 6.0 | 14.4724 | 14.226 | 69.0549 | 1.89565 | 1.99641 | 21.8022 | 103.7 | 27.2478 | 0.193265 | 1.6825 | 0.0773059 | 5.85819 | 0 |
| 7.0 | 8.86817 | 10.9863 | 68.7655 | 1.81999 | 1.99381 | 21.7437 | 103.7 | 27.2463 | 0.333371 | 2.90222 | 0.133348 | 10.105 | 0 |
| 8.0 | 0 | 5.45333 | 68.2714 | 1.69079 | 1.98936 | 21.6437 | 103.7 | 27.2436 | 0.572648 | 4.9853 | 0.229059 | 17.358 | 0 |
| 9.0 | 0 | 5.45333 | 68.2714 | 1.69079 | 1.98936 | 21.6437 | 103.7 | 27.2436 | 0.572648 | 4.9853 | 0.229059 | 17.358 | 0 |

## Visualizing actual vs predicted isoprenol concentrations for ART's recommendations¶

Collect all isoprenol production values in a single list first:

In [15]:

```
production = []
for i in range(user_params['numinstances']):
    cell, Emets = cellsEmetsBE[i]
    production.append(Emets.loc[9,'isoprenol_e'])
```

### Creating a new data frame with production information¶

In [16]:

```
production_df = rec_df.copy()
production_df['Actual Isoprenol [mM]'] = production.copy()
production_df.loc[0:2,:]
```

Out[16]:

|  | Line Name | ACCOAC | MDH | PTAr | CS | ACACT1r | PPC | PPCK | PFL | Mean predicted Isoprenol [mM] | SD Isoprenol [mM] | Actual Isoprenol [mM] |
| --- | --- | --- | --- | --- | --- | --- | --- | --- | --- | --- | --- | --- |
| 0 | Strain 97 | 2.0 | 2.0 | 1.0 | 2.0 | 2.0 | 2.0 | 0.0 | 1.0 | 0.573584 | 0.019785 | 0.572648 |
| 1 | Strain 98 | 2.0 | 2.0 | 2.0 | 2.0 | 2.0 | 2.0 | 0.0 | 1.0 | 0.572193 | 0.019752 | 0.572648 |
| 2 | Strain 99 | 1.0 | 2.0 | 1.0 | 2.0 | 2.0 | 2.0 | 0.0 | 1.0 | 0.572167 | 0.019752 | 0.572648 |

### Plotting predicted vs actual production:¶

In [17]:

```
pred_vs_actual(production_df)
```

### Saving dataframe as csv¶

In [18]:

```
df_filename = '../data/ART_recommendations_with_production.csv'
production_df.to_csv(df_filename)
```

## Generate omics data for the best recommendation¶

We will generate omics data for the best recommendation, and store it in EDD. Firstly, we obtain the multiomics data for each time point:

In [19]:

```
proteomics_timeseries = {}
transcriptomics_timeseries = {}
metabolomics_timeseries = {}
metabolomics_oldids_timeseries = {}
fluxomics_timeseries = {}
        
for t in tspan:
    fluxomics_timeseries[t] = solutionsMOMA_TS[0][t].fluxes.to_dict()
    (proteomics_timeseries[t], transcriptomics_timeseries[t], 
     metabolomics_timeseries[t], metabolomics_oldids_timeseries[t]) = omg.get_multiomics(model, 
                                                                                         solutionsMOMA_TS[0][t], 
                                                                                         user_params['mapping_file'], 
                                                                                         old_ids=True)
```

### Writing the multiomics data, cell concentration and external metabolites concentration data into output files¶

#### EDD data output¶

Write experiment description:

In [20]:

```
omg.write_experiment_description_file(user_params['edd_omics_file_path'], line_name='Strain 97', label='_BT')
```

Write OD data:

In [21]:

```
omg.write_OD_data(cell, user_params['edd_omics_file_path'], line_name='Strain 97', label='_BT')
```

Write external metablites:

In [22]:

```
omg.write_external_metabolite(Emets, user_params['edd_omics_file_path'], line_name='Strain 97', label='_BT')
```

Write omics data:

In [23]:

```
omg.write_omics_files(fluxomics_timeseries, 'fluxomics', user_params, line_name='Strain 97', label='_BT')
omg.write_omics_files(proteomics_timeseries, 'proteomics', user_params, line_name='Strain 97', label='_BT')
omg.write_omics_files(transcriptomics_timeseries, 'transcriptomics', user_params, line_name='Strain 97', label='_BT')
omg.write_omics_files(metabolomics_timeseries, 'metabolomics', user_params, line_name='Strain 97', label='_BT')
```

We will also write a small version of the multiomics data with a subset of proteins, transcripts and metabolites:

In [24]:

```
genesSM       = ['b0180','b2708','b3197','b1094','b2224','b3256','b2316','b3255','b0185','b1101'] 
proteinsSM    = ['P17115','P45395','P0A6A8','P76461','P77580','P24182','P0A9Q5','P0ABD5','P77580','P00893']
metabolitesSM = ['CID:1549101','CID:175','CID:164533','CID:15938965','CID:21604863','CID:15939608','CID:27284','CID:1038','CID:16741146','CID:1778309']
```

In [25]:

```
transcriptomics_timeseriesSM ={}
proteomics_timeseriesSM      ={}
metabolomics_timeseriesSM    ={}
for t in tspan:
    transcriptomics_timeseriesSM[t] = {gene:    transcriptomics_timeseries[t][gene] for gene    in genesSM}
    proteomics_timeseriesSM[t]      = {protein: proteomics_timeseries[t][protein]   for protein in proteinsSM}
    metabolomics_timeseriesSM[t]    = {metab:   metabolomics_timeseries[t][metab]   for metab   in metabolitesSM}
```

In [26]:

```
omg.write_omics_files(proteomics_timeseriesSM,     'proteomics'     , user_params, line_name='Strain 97', label='_BTSM')
omg.write_omics_files(transcriptomics_timeseriesSM,'transcriptomics', user_params, line_name='Strain 97', label='_BTSM')
omg.write_omics_files(metabolomics_timeseriesSM,   'metabolomics'   , user_params, line_name='Strain 97', label='_BTSM')
```

In [ ]:

```

```
